# Synthesis and Characterization of a pH- and Temperature-Sensitive Fe_3_O_4_-SiO_2_-Poly(NVCL-co-MAA) Nanocomposite for Controlled Delivery of Doxorubicin Anticancer Drug

**DOI:** 10.3390/polym15040968

**Published:** 2023-02-15

**Authors:** Jorge Luis Sánchez-Orozco, Héctor Iván Meléndez-Ortiz, Bertha Alicia Puente-Urbina, Oliverio Santiago Rodríguez-Fernández, Antonia Martínez-Luévanos, Luis Alfonso García-Cerda

**Affiliations:** 1 Departamento de Materiales Avanzados, Centro de Investigación en Química Aplicada, Blvd. Enrique Reyna Hermosillo #140, Saltillo 25294, Mexico; 2 CONACyT, Centro de Investigación en Química Aplicada, Blvd. Enrique Reyna Hermosillo #140, Saltillo 25294, Mexico; 3 Facultad de Ciencias Químicas, Universidad Autónoma de Coahuila, Blvd. V. Carranza s/n, Saltillo 25280, Mexico

**Keywords:** magnetic nanocomposite, stimulus sensitive, PNVCL, PMAA, drug release

## Abstract

This work reports the synthesis, characterization, and in vitro release studies of pH- and temperature-sensitive Fe_3_O_4_-SiO_2_-poly(NVCL-co-MAA) nanocomposite. Fe_3_O_4_ nanoparticles were prepared by chemical coprecipitation, coated with SiO_2_ by the Stöber method, and functionalized with vinyl groups. The copolymer poly(N-vinylcaprolactam-co-methacrylic acid) (poly(NVCL-co-MAA)) was grafted onto the functionalized Fe_3_O_4_-SiO_2_ nanoparticles by free radical polymerization. XRD, FTIR, TGA, VSM, and TEM techniques were performed to characterize the nanocomposite. The release behavior of Doxorubicin (DOX) loaded in the nanocomposite at pH 5.8 and 7.4, and two temperatures, 25 and 37 °C, was studied. According to the release studies, approximately 55% of DOX is released in 72 h at pH 7.4, regardless of temperature. At pH 5.8, 78% of DOX was released in 48 h at 25 °C, and when increasing the temperature to 37 °C, more than 95 % of DOX was released in 24 h. The DOX release data treated with Zero-order, first-order, Higuchi, and Korsmeyer–Peppas models showed that Higuchi’s model best fits the data, indicating that the DOX is released by diffusion. The findings suggest that the synthesized nanocomposite may be useful as a DOX carrier in biomedical applications.

## 1. Introduction

In recent years, magnetic nanocomposites have received much attention due to their physical, chemical, and biological properties and, further, their broad biomedical applications in drug-delivery systems [1,2,3,4], hyperthermia [5,6,7], magnetic resonance [8,9,10], and others [11]. Due to their excellent biocompatibility and high loading capacity, these nanocomposites are ideal for fabricating drug-delivery vehicles. They focused on the body by using a magnet, reaching the area of interest, and releasing the bioactive agent in a controlled manner, thus improving the therapeutic effects in patients [12]. The administration of drugs generally must be specific and focused on the damaged area since the most commonly used anticancer drugs cause unwanted side effects in healthy cells [13]. Doxorubicin (DOX) is a traditional chemotherapeutic agent used to treat breast, prostate, and gastric cancer [14,15]. However, it has limited use due to its toxicity and strong side effects.

Magnetite (Fe_3_O_4_) is the most commonly used magnetic material for medical applications due to its good biocompatibility and biodegradability; in addition, its surface is easily modified with polymers [16,17,18] and inorganic compounds [19,20,21]. The grafting of silica coatings or biocompatible stimuli-responsive polymers onto the surface of Fe_3_O_4_ nanoparticles offers good physico-chemical properties. A mesoporous silica coating enhances the chemical and thermal stability of the Fe_3_O_4_ nanoparticles and facilitates the grafting of polymers on their surface due to the presence of silanol groups [22,23,24]. However, the Fe_3_O_4_-SiO_2_ system has limited drug loading and a lack of stimuli-responsive release [25].

Tumor tissue is known for having lower pH (5.5–6) and higher temperature (40 °C) than normal tissue. Therefore, drug-release systems capable of releasing anti-cancer actives in response to a physiological change, such as temperature and pH, could be good candidates for treating tumor cells and minimizing toxicity into healthy cells [26]. Temperature and pH-sensitive magnetic nanocarriers are interesting stimuli-sensitive systems to be used in biomedical applications [27].

Recently, the synthesis of stimuli-sensitive polymers has been investigated to obtain copolymers with a thermal and pH response for drug-delivery systems [28,29]. A slight increase in temperature or pH variation will promote a phase transition of polymer chains (from hydrophilic to hydrophobic or vice-versa), resulting in the release of the loaded drug [26]. Poly(N-vinylcaprolactam) (PNVCL) is a biocompatible and thermo-responsive polymer, and its lower critical solution temperature (LCST) is in the range of 33–35 °C, close to body temperature (37 °C) and, consequently, it could be used in drug-delivery systems and biomedical applications [30,31,32]. On the other hand, poly(methacrylic acid) (PMAA) is a pH-sensitive polymer that can change its conformational state at pH values from 5.5 to 6. Polymer chains expand at high pH values, but at low pH values, they contract due to the electrostatic repulsion of charged carboxyl groups [33].

Different research groups have reported the preparation of anticancer drug nanocarriers of magnetic and SiO_2_ nanoparticles mixed with stimulus-sensitive polymers [34,35]. For example, Mdlovu et al. prepared nanocarriers by modifying magnetic iron oxide nanoparticles with crosslinked Pluronic F127 and branched polyethyleneimine [36]. They carried out DOX loading and release studies at pH 7.4 and 5.4 and temperatures of 37 and 42 °C, observing that drug release was dependent on both pH and temperature, obtaining a maximum release of 55% of DOX at pH 5.4/37 °C in 48 h, and when the temperature increases from 37 to 42 °C, the release of DOX increased by 11%. Furthermore, Mandal and co-workers prepared a triple-responsive (pH, magnetic field, and redox) nanogel for the controlled delivery of DOX by trapping magnetic nanoparticles in a pentaerythritol-poly(ε-caprolactone)-b-poly(acrylic acid) copolymer [37]. The nanogels showed promising DOX loading and release behavior, with a maximum DOX release value of 65% under the synergistic participation of all three stimuli.

Thus, this work aims to synthesize and characterize a Fe_3_O_4_-SiO_2_-poly(NVCL-co-MAA) nanocomposite with a dual response and study its ability to load and release DOX in a controlled manner. This nanocomposite responds to changes in pH and temperature, which allows it to act selectively on specific areas of the body.

## 2. Materials and Methods

### 2.1. Chemicals

All reagents were of analytical grade and used as received without any purification: Ferric chloride hexahydrate (FeCl_3_.6H_2_O, 97%), ferrous chloride tetrahydrate (FeCl_2_.4H_2_O, 99%), ammonium hydroxide (NH_4_OH_(ag)_, 28–30%), tetraethylorthosilicate (TEOS, 98%), vinyltrimethoxysilane (VTMS, 98%), toluene (99.9%), magnesium sulfate (MgSO_4_, 99.5%), N-vinylcaprolactam (NVCL, 98%), methacrylic acid (MAA, 99%), ammonium persulfate (APS, 98%), N, N’-Methylenebisacrylamide (NMBS, 99%), Doxorubicin hydrochloride (DOX, 98–102%) Alsever´s solution, and phosphate buffered saline solution (PBS, pH 7.4) were purchased from Sigma-Aldrich (St. Louis, MO, USA). Ethanol (99%) and isopropyl alcohol (99%) were purchased from JT Baker (Phillipsburg, NJ, USA).

### 2.2. Preparation of Fe_3_O_4_ Nanoparticles (MNPsFe_3_O_4_)

The preparation of MNPsFe_3_O_4_ was carried out via the chemical coprecipitation method as follows: FeCl_3_·6H_2_O and FeCl_2_·4H_2_O in a proportion of 2:1 (wt/wt) were mixed in 350 mL of deionized water under mechanical stirring (3000 rpm). Subsequently, the solution was heated at 70 °C, and the stirring speed was increased to 7000 rpm, achieving coprecipitation by adding 100 mL of NH_4_OH_(aq)_. The MNPsFe_3_O_4_ obtained were separated by decantation using a magnet and washed with a solution of ethanol and deionized water (50:50 *v/v*).

### 2.3. Coating of SiO_2_ onto Fe_3_O_4_ Nanoparticles (MNPsFe_3_O_4_-SiO_2_)

The Fe_3_O_4_ nanoparticles were coated with SiO_2_ using the Stöber method [19]: MNPsFe_3_O_4_ (300 mg) was added to a mixture of isopropyl alcohol (50 mL) and deionized water (4 mL) and dispersed by sonication for 20 min. Then, 5 mL of NH_4_OH was added slowly to the solution until it reached a pH of 10.5. Next, 2 mL of TEOS was added dropwise and stirred for 24 h. The obtained nanoparticles were washed with deionized water and ethanol (50:50 *v/v*). The remaining MNPsFe_3_O_4_-SiO_2_ was separated using a magnet and then dried at 80 °C for subsequent use.

### 2.4. Functionalization of Fe_3_O_4_-SiO_2_ Nanoparticles with VTMS (MNPsFe_3_O_4_-SiO_2_-VTMS)

The functionalization of MNPsFe_3_O_4_-SiO_2_ was carried out according to our previous works [38,39]. Briefly, VTMS (300 mg) was dissolved in 13 mL of toluene (previously dehydrated with MgSO_4_) under constant stirring for 30 min. Then, MNPsFe_3_O_4_-SiO_2_ (150 mg) was added and sonicated for 20 min. The resulting dispersion was heated at a reflux temperature (110 °C) under magnetic stirring (400 rpm) for 24 h. After this time, the nanoparticles were recovered magnetically and rinsed with toluene.

### 2.5. Synthesis of Fe_3_O_4_-SiO_2_-Poly(NVCL-co-MAA) Nanocomposite

The nanocomposite was synthesized by free radical polymerization: First, 450 mg of NVCL and 50 mg of MAA (9:1 ratio) were dissolved in 15 mL of deionized water. Then, 50 mg of APS (initiator) and 50 mg of NMBS (crosslinking agent) were added to the solution. After that, we added 150 mg of MNPsFe_3_O_4_-SiO_2_-VTMS to the solution and sonicated it for 20 min. The resulting dispersion was degassed by N_2_ bubbling and then polymerized at 60 °C for 24 h. Finally, the synthesized nanocomposite was rinsed five times using deionized water under vigorous stirring to remove either the residual monomer or non-grafted copolymer.

### 2.6. Characterization

X-ray diffraction (XRD) patterns were collected in a Rigaku Ultima IV diffractometer (Rigaku Corporation, Akishima-shi, Tokyo, Japan) from 10 to 80° (2θ). The Fourier transform infrared (FTIR) spectra were collected in a Nicolet Magna 550 FTIR spectrophotometer (Nicolet Instrument Corporation, Madison, WI, USA) in the wavenumber range of 4000 to 400 cm^−1^. Thermogravimetric analysis was performed in a TGA Q500 thermal analyzer (TA Instruments, New Castle, DE, USA), and the samples were heated at 10 °C min^−1^ from room temperature up to 800 °C in a nitrogen atmosphere. The magnetic measurements were performed at room temperature using a SQUID magnetometer PPMS 6000 (Quantum Design, San Diego, CA, USA) under a magnetic field of -20 to 20 kOe. The morphology and size of the obtained materials were examined using a high-resolution transmission electron microscope (HRTEM, FEI Titan 80–300) (FEI Company, Hillsboro, OR, USA). The critical solution temperature (LCST) and pH response of the nanocomposite were determined by dynamic light scattering (DLS) using a Nanotrac Wave II (Microtrac Inc., York, PA, USA).

### 2.7. Loading of DOX onto MNPsFe_3_O_4_-SiO_2_ and MNPsFe_3_O_4_-SiO_2_-Poly(NVCL-co-MAA)

For DOX loading, 25 mg of the sample was sonicated in 5 mL of a solution containing 0.1 mg of DOX/mL of water and kept under magnetic stirring for 72 h at room temperature in darkness. The DOX-loaded sample was separated from the solution using a magnet, washed with deionized water, and dried. The amount of free DOX was calculated by measuring the supernatant solution using UV-Vis spectroscopy at 481 nm. The experiments were performed in triplicate. The DOX loaded (DL) was calculated as follows:

(1) $$\mathrm{DL}=\frac{\left(C_{0}-C_{t}\right)\;V}{m},$$

where *C*_0_ (mg/mL) is the initial DOX concentration in solution and *C_t_* is the concentration of DOX at time *t*, *V* (mL) is the volume of the DOX solution, and *m* (g) is the mass of the nanocomposite (NC).

### 2.8. In Vitro DOX Release Studies

The release studies were carried out at four different conditions to evaluate the pH and temperature sensitivity of the nanocomposite. The release studies were performed at pH 5.8 and 7.4 and at two different temperatures (25 and 37 °C). In each study, samples previously loaded with DOX were redispersed in 1 mL of PBS and placed into a dialysis membrane (MWCO 14,000). The membrane was firmly tied at both ends with nylon thread and immersed in 30 mL of PBS at the studied temperature under magnetic stirring. The release of DOX was measured by UV-Vis spectroscopy at 481 nm. The experiments were performed in triplicate. The cumulative percentage of DOX released was calculated as follows:

(2) $$\mathrm{Cumulative}\;\mathrm{drug}\;\mathrm{released}\;\left(\%\right)=\frac{Q_{t}}{Q_{0}}\times 100,$$

where *Q_t_* is the amount of DOX released at time t and *Q*_0_ is the amount of DOX loaded in the nanocomposite.

### 2.9. In Vitro Hemolysis Assay

The hemolysis assay was conducted using human blood from non-smokers and healthy donors according to the ASTM-F756-13 standard method [40]. The blood was collected in 10 mL vacutainer tubes with EDTA (anticoagulant) and centrifuged at 4000 rpm for 5 min at 5 °C. After that, the supernatant (plasma) was discarded, and the precipitate (erythrocytes) was washed with a cold Alsever solution (5 times). A dilution of erythrocytes was made by mixing 100 μL of erythrocytes in 10 mL of Alsever´s solution. Then, 150 μL of the erythrocyte dilution and 1850 μL of Alsever´s solution was mixed with 10, 50, 125, 250, and 500 μg/mL of the nanocomposite. The erythrocyte dilution with deionized water and Alsever´s solution were used as positive and negative controls, respectively. The resulting suspensions were incubated at 37 °C for 24 h and centrifuged at 4000 rpm. The absorbance of the supernatant was measured in a UV-Vis spectrophotometer at 415 nm (Spectronic Instruments Genesys 5). The hemolysis percentage was calculated as follows:

(3) $$\mathrm{Hemolysis}\;\left(\%\right)=\frac{\left(A_{\mathrm{sample}}-A_{c-}\right)}{\left(A_{C+}-A_{C-}\right)}\times 100,$$

where *A_C_*_−_ and *A_C_*_+_ represent the absorbance of negative and positive controls, respectively. All trials were carried out in triplicate.

### 2.10. Release Kinetic Models

In order to study the DOX release mechanism, experimental data were fitted to four kinetic models. The zero-order model describes the drug release of dosage forms that help to maintain constant levels of the active drug in the blood during the entire delivery period (Equation (4)). The first-order model (Equation (5)) describes the drug release where the release rate depends on the concentration. The Higuchi model describes the active agent release from solid and semi-solid matrices (Equation (6)). Finally, the Korsmeyer–Peppas model (Equation (7)) describes the release of drugs from polymer matrices, analyzing the type of diffusion.

(4) $$Q_{t}=Q_{0}K_{0}t$$

(5) $$\ln Q_{t}=\ln Q_{0}+K_{1}t$$

(6) Qt=KHt12

(7) $$\frac{Q_{t}}{Q_{\infty }}=K_{k}t^{\eta }$$

where *Q_t_* is the amount of drug released at time *t*, *Q*_0_ is the initial amount of drug in the solution, *K*_0_, *K_H_*, and *K_k_* are the constants of the equations, *Q_t_/Q*_∞_ is the fraction of the drug released at time *t*, and *η* is the drug release exponent. Values of 0.45 ≤ *η* correspond to Fickian type diffusion mechanism, 0.45 < *η* > 0.89 to non-Fickian transport, *η* = 0.89 to Case II transport, and *η* > 0.89 to Super Case II transport [41,42,43]. The best-fitting model was identified according to the correlation coefficient (R^2^).

## 3. Results and Discussion

### 3.1. Synthesis of MNPsFe_3_O_4_-SiO_2_-g-Poly(NVCL-co-MAA) Nanocomposites

**Figure 1:**
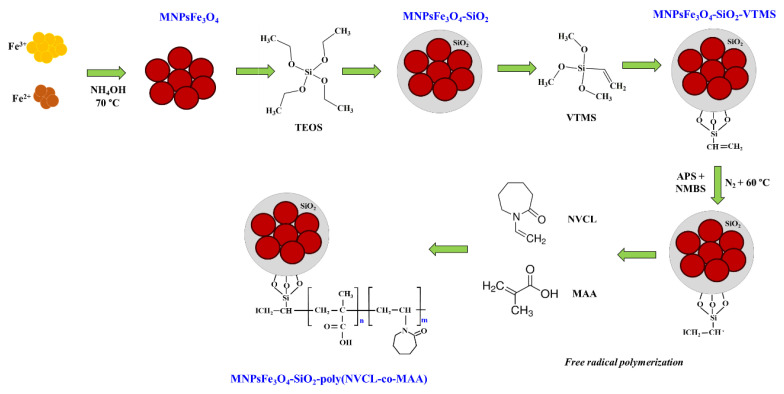
Synthesis route of MNPsFe_3_O_4_-SiO_2_-poly(NVCL-co-MAA).

First, the MNPsFe_3_O_4_ was synthesized using the chemical coprecipitation method consisting of FeCl_3_ and FeCl_2_ as iron sources and an aqueous NH_4_OH solution as a precipitant agent. The obtained MNPsFe_3_O_4_ were coated with SiO_2_ using the Stöber method, which consists of the hydrolysis and polycondensation of TEOS onto the magnetic nanoparticles. Then, the MNPsFe_3_O_4_-SiO_2_ was functionalized with VTMS to anchor free vinyl groups on the surface to perform the radical polymerization reaction. Finally, the copolymer Poly(NVCL-co-MAA) was grafted onto the MNPsFe_3_O_4_-SiO_2_-VTMS through free radical polymerization (Figure 1).

### 3.2. Characterization

**Figure 2:**
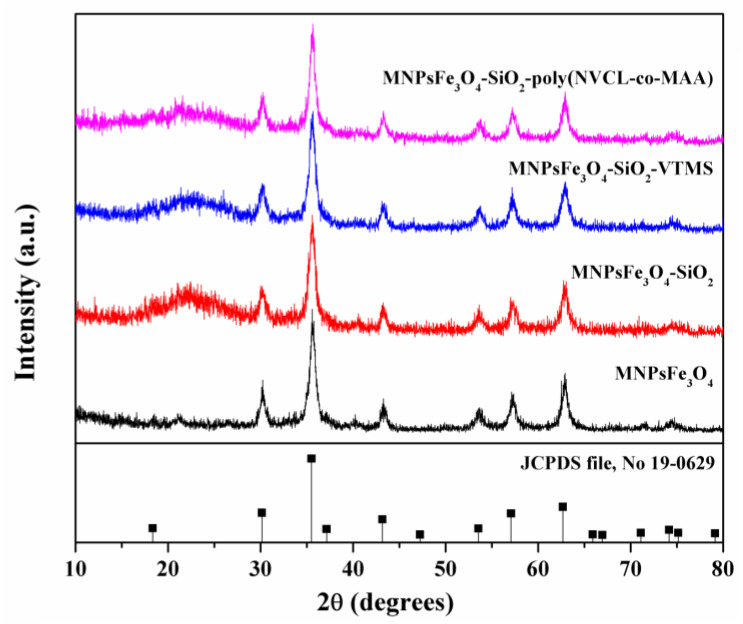
XRD patterns of MNPsFe_3_O_4_-SiO_2_, MNPsFe_3_O_4_-SiO_2_-VTMS, and MNPsFe_3_O_4_-SiO_2_-poly (NVCL-co-MAA) nanocomposites.

XRD patterns of MNPsFe_3_O_4_, MNPsFe_3_O_4_-SiO_2_, MNPsFe_3_O_4_-SiO_2_-VTMS, and the nanocomposite of MNPsFe_3_O_4_-SiO_2_-poly(NVCL-co-MAA) are shown in Figure 2. Seven characteristic peaks were identified for all samples and located at 2θ values of 30.09, 35.44, 43.05, 53.39, 56.94, 62.51, and 73.94°. These peaks correspond to crystalline planes with hkl indices: (220), (311), (400), (422), (511), (440), and (533), respectively, corroborating the presence of a magnetite spinel-type structure according to the standard (JCPDS file, No. 19-0629). Regarding samples modified with SiO_2_ and VTMS, the corresponding XRD patterns observed no relevant changes in the crystalline structure. However, a broad peak can be observed from 15 to 30°, corresponding to the amorphous silica. On the other hand, the XRD pattern of MNPsFe_3_O_4_-SiO_2_-poly(NVCL-co-MAA) does not show secondary phases or changes in the crystal structure of MNPsFe_3_O_4_ [44].

**Figure 3:**
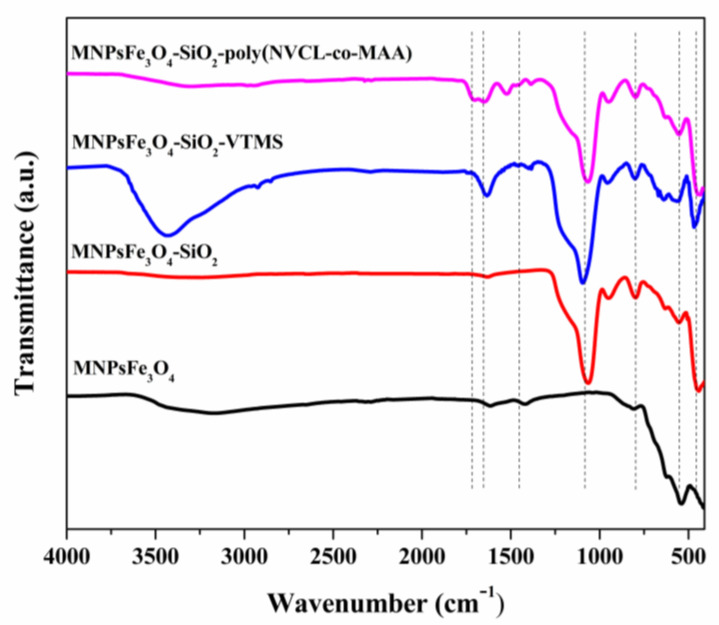
FTIR spectra of MNPsFe_3_O_4_-SiO_2_, MNPsFe_3_O_4_-SiO_2_-VTMS, and MNPsFe_3_O_4_-SiO_2_-poly (NVCL-co-MAA) nanocomposites.

Figure 3 shows the FTIR spectra for MNPsFe_3_O_4_, MNPsFe_3_O_4_-SiO_2_, MNPsFe_3_O_4_-SiO_2_-VTMS, and MNPsFe_3_O_4_-SiO_2_-poly(NVCL-co-MAA). The FTIR spectrum of MNPsFe_3_O_4_ shows a band at 560 cm^−1^ related to the Fe-O bending vibration. After modification with SiO_2_, the sample of MNPsFe_3_O_4_-SiO_2_ shows a band at 475 cm^−1^ corresponding to the Fe-O-Si stretching vibration and two bands located at 795 cm^−1^ and 1060 cm^−1^ associated with the bending and stretching of the Si-O-Si bond, respectively. The sample of MNPsFe_3_O_4_-SiO_2_-VTMS shows an additional low-intensity band at 1450 cm^−1^ related to the vinyl group (C=C) from VTMS [45,46]. The FTIR spectrum of MNPsFe_3_O_4_-SiO_2_-poly(NVCL-co-MAA) shows one band at 1637 cm^−1^ due to the C=O stretching vibration of the amide group from PNVCL, while the band at 1482 cm^−1^ can be assigned to the C-N stretching vibration. Moreover, we can observe a band at 1716 cm^−1^ due to the vibration of the C=O bond of the carboxylic acid groups (-COOH) from PMAA in the copolymer [47,48]. The FTIR spectra of MNPsFe_3_O_4_-SiO_2_-poly(NVCL-co-MAA) with different monomer ratios (NVCL:MAA) are presented in Figure S1 (Supplementary Materials).

**Figure 4:**
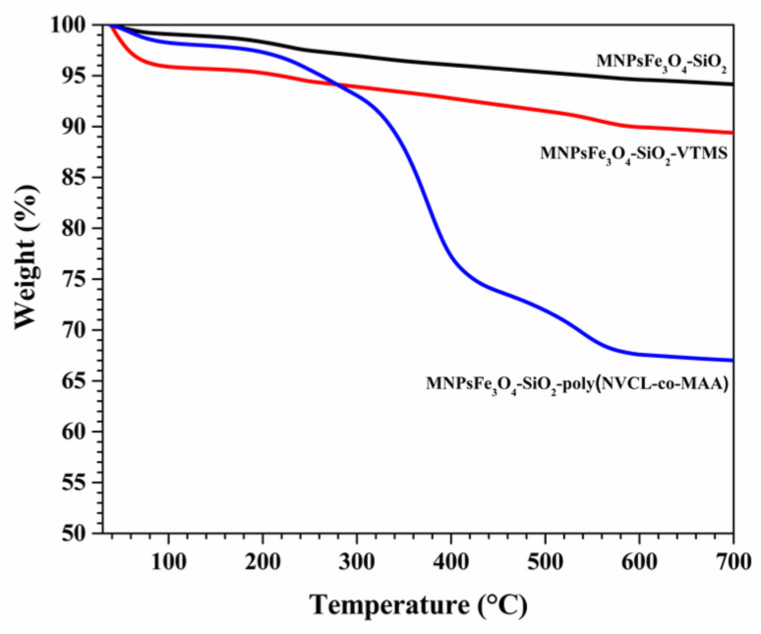
TGA curves of MNPsFe_3_O_4_-SiO_2_, MNPsFe_3_O_4_-SiO_2_-VTMS, and MNPsFe_3_O_4_-SiO_2_-poly (NVCL-co-MAA) nanocomposites.

TGA curves of MNPsFe_3_O_4_-SiO_2_, MNPsFe_3_O_4_-SiO_2_-VTMS, and the nanocomposite of MNPsFe_3_O_4_-SiO_2_-poly(NVCL-co-MAA) are shown in Figure 4. The TGA curve for MNPsFe_3_O_4_-SiO_2_ showed a total weight loss of 7% in two events. The first event, from room temperature to 200 °C, may be attributed to the evaporation of adsorbed water from the sample, and the second event between 200 and 300 °C is characteristic of the decomposition of the hydroxyl groups from the silica. The TGA curve of MNPsFe_3_O_4_-SiO_2_-VTMS shows a weight loss of 3% in the temperature range of 40–200 °C due to the evaporation of physisorbed water and another of 8% due to the decomposition of VTMS (200–600 °C). [49]. On the other hand, the thermal degradation of the nanocomposite of MNPsFe_3_O_4_-SiO_2_-poly(NVCL-co-MAA) shows a total weight loss of 33% in three events: 200–280 °C, 320–420 °C, and after 430 °C. The first event is related to the anhydration of PMAA, and the second event can be assigned to the random scission of PMAA chains, while the third is due to the thermal decomposition of NVCL [50]. A quantitative analysis of TGA data indicates that the content of VTMS in the MNPsFe_3_O_4_-SiO_2_-VTMS was 8%, and the grafted copolymer in the nanocomposite was 33%.

**Figure 5:**
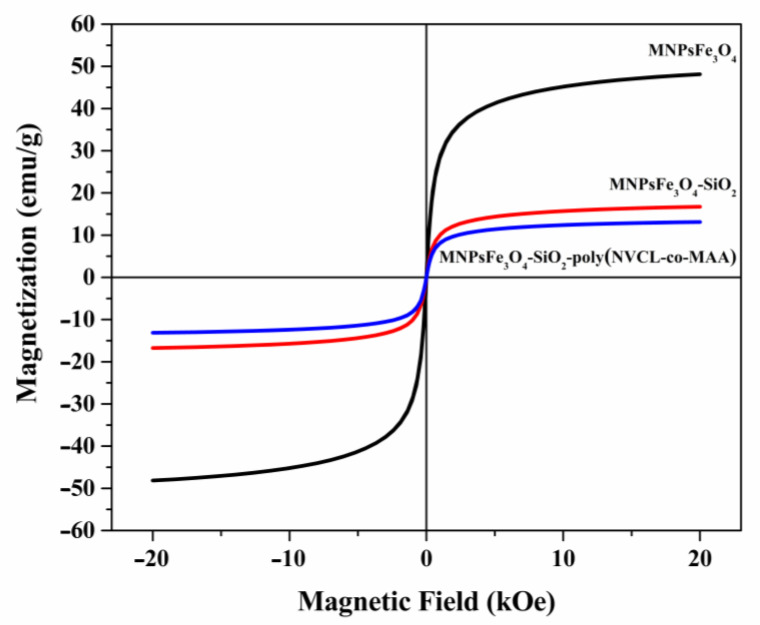
Magnetization curves of MNPsFe_3_O_4_, MNPsFe_3_O_4_-SiO_2_, and MNPsFe_3_O_4_-SiO_2_-poly (NVCL-co-MAA) nanocomposites.

**Figure 6:**
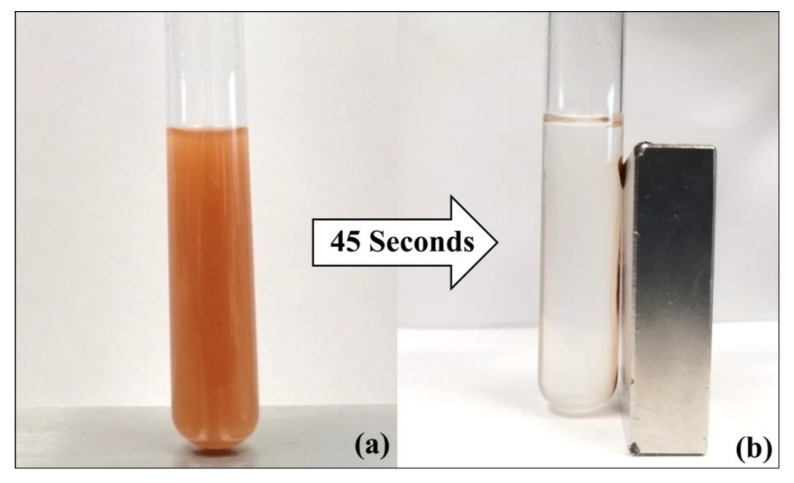
MNPsFe_3_O_4_-SiO_2_-poly (NVCL-co-MAA), before (**a**) and after 45 s (**b**) of the application of a magnetic field.

The magnetization curves for MNPsFe_3_O_4_, MNPsFe_3_O_4_-SiO_2_, and MNPsFe_3_O_4_-SiO_2_-poly(NVCL-co-MAA) nanocomposites are shown in Figure 5. The magnetization values have been normalized to the total mass of the sample. All the samples exhibited low values of coercivity (<5 Oe) and remanence (<1 emu/g), suggesting a superparamagnetic behavior. The saturation magnetization (Ms) values for MNPsFe_3_O_4_, MNPsFe_3_O_4_-SiO_2_, and MNPsFe_3_O_4_-SiO_2_-poly(NVCL-co-MAA) were 48.1, 16.7, and 13.1 emu/g, respectively. The decrease in the Ms value of MNPsFe_3_O_4_ compared to other samples is due to the presence of non-magnetic materials (silica and polymer) [51]. Despite the decrease in Ms, all the materials are easily separable using a magnet (Figure 6).

**Figure 7:**
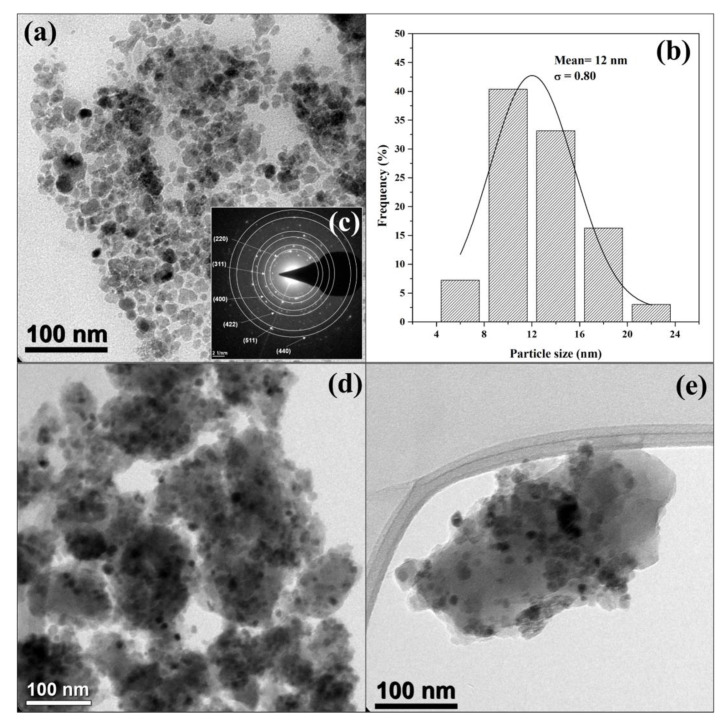
TEM image (**a**), particle size histogram (**b**), and SAED pattern (**c**) of Fe_3_O_4_ nanoparticles, TEM image of MNPsFe_3_O_4_-SiO_2_ (**d**) and MNPsFe_3_O_4_-SiO_2_-poly (NVCL-co-MAA) nanocomposite (**e**).

The MNPsFe_3_O_4_ showed a semi-spherical morphology with some aggregates due to their magnetic nature (Figure 7a). According to the histogram in Figure 7b, the nanoparticles showed sizes between 4 and 24 nm. The solid black line corresponds to the log-normal distribution fit and gives an average particle size of 12 nm and a standard deviation (σ) of 0.80. The selected area electron diffraction (SAED) pattern (Figure 7c) of MNPsFe_3_O_4_ shows six rings corresponding to the crystalline planes with hkl indices (220), (311), (400), (422), (511), and (440). On the other hand, the TEM micrograph for MNPsFe_3_O_4_-SiO_2_ (Figure 7d) showed nanoparticles of MNPsFe_3_O_4_ embedded within the SiO_2_ matrix, confirming the formation of this material [46,52]. The micrograph for MNPsFe_3_O_4_-SiO_2_ -poly (NVCL-co-MAA) (Figure 7e) showed the presence of nanoparticles embedded within a matrix of silica and the copolymer.

**Figure 8:**
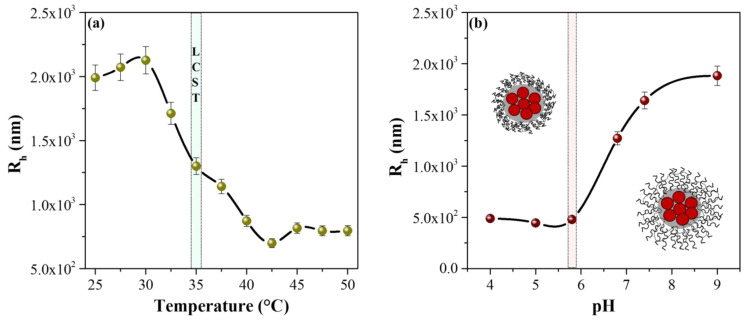
LCST (**a**) and pH response (**b**) of MPNsFe_3_O_4_-SiO_2_-poly(NVCL-co-MAA)_33%_ nanocomposite.

The LCST of the MPNsFe_3_O_4_-SiO_2_-poly(NVCL-co-MAA) nanocomposite was measured by dynamic light scattering and the results are shown in Figure 8a. The nanocomposite’s hydrodynamic radius (Rh) variation was investigated at increasing temperatures from 25 to 50 °C, with a heating interval of 2.5 °C and a fixed concentration of the nanocomposite (1 mg/mL in water). According to the results, the nanocomposite exhibited an LCST of 35 °C. We can observe an increase in the Rh below this temperature due to the formation of hydrogen bonds between PNVCL chains and the water molecules. Meanwhile, above this temperature, a decrease in Rh was observed since the hydrophobic interactions of the PNVCL chains predominated, which caused their total collapse. Therefore, the hydrophilic–hydrophobic phase transition of PNVCL chains causes a turning point in the curve identified as the LCST of the MPNsFe_3_O_4_-SiO_2_-poly(NVCL-co-MAA) nanocomposite [53,54,55].

Furthermore, using DLS, the variation of the hydrodynamic radius of the MPNsFe_3_O_4_-SiO_2_-poly(NVCL-co-MAA) nanocomposite was investigated as a function of pH, and the results are presented in Figure 8b. As can be seen, the Rh of the nanocomposites is strongly influenced by the pH of the medium, presenting a phase transition at pH 5.8, which is close to the pKa of PMAA (pKa of 5.5). This phase transition is primarily due to the protonation-deprotonation of the carboxylic acid group of the PMAA chains, which is protonated in the presence of an aqueous solution with a pH lower than pKa (5.5). Therefore, PMAA chains tend to form hydrogen bonds towards the interior of the polymeric structure, which induces the formation of a collapsed structure. However, as the pH of the medium is increased from 5.8 to 9, the COOH groups ionize, and the PMAA chains become hydrophilic, reaching an elongated conformation and increasing the Rh [56,57,58]. The obtained nanocomposites showed a dual response to pH and temperature from these results, which could help to develop new drug nanocarriers.

### 3.3. DOX Loading

**Figure 9:**
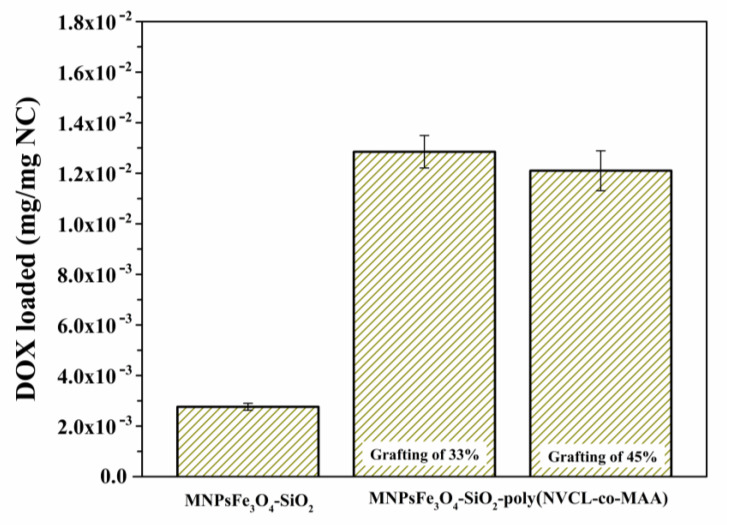
DOX loading capability of MNPsFe_3_O_4_-SiO_2_ and MNPsFe_3_O_4_-SiO_2_-poly(NVCL-co-MAA)_33.45%_ nanocomposites.

Figure 9 shows the DOX loading capability of MNPsFe_3_O_4_-SiO_2_ and MNPsFe_3_O_4_-SiO_2_-poly(NVCL-co-MAA) nanocomposites containing different percentages of the copolymer (33 and 45%). The MNPsFe_3_O_4_ failed to load DOX, while MNPsFe_3_O_4_-SiO_2_ loaded 2.76 × 10^−3^ mg DOX/mg of NC. On the other hand, the nanocomposites of MNPsFe_3_O_4_-SiO_2_-poly(NVCL-co-MAA) with 33 and 45% grafting present similar loads, 1.29 × 10^−2^ and 1.2 × 10^−2^ mg DOX/mg of NC, respectively. It can be noted that the amount of loading DOX was higher for the MNPsFe_3_O_4_-SiO_2_-poly(NVCL-co-MAA) nanocomposites compared to the MNPsFe_3_O_4_-SiO_2_. DOX is readily loaded into the nanocomposite due to the strong interaction between carboxylic groups from PMAA and the amino groups from DOX [59,60]. However, DOX loading values were slightly reduced by increasing the poly(NVCL-co-MAA) grafting fraction on MNPsFe_3_O_4_-SiO_2_. This behavior could be due to the higher content of polymer grafting on the MNPsFe_3_O_4_-SiO_2_, which makes it more difficult for DOX molecules to penetrate the polymeric network of the nanocomposite, limiting drug loading [34].

### 3.4. DOX Release

**Figure 10:**
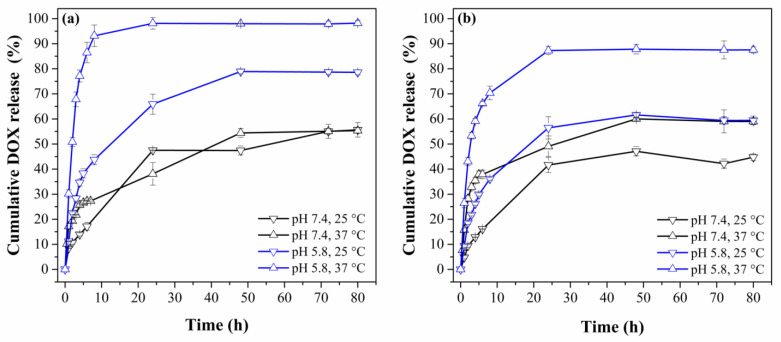
Cumulative DOX release profiles of MNPsFe_3_O_4_-SiO_2_-poly (NVCL-co-MAA) nanocomposites with 33% (**a**) and 45% (**b**) grafting.

DOX release profiles are shown in Figure 10 and Figure S2. The MNPsFe_3_O_4_-SiO_2_ exhibited a maximum release of 37% (at pH 7.4 and 37 °C) (Figure S2). This low percentage may be because only the DOX interacting with the surface of the SiO_2_ matrix is released. At the same time, the drug loaded within the silica coating cannot be released due to strong interactions by forming hydrogen bonds between the silanol groups (Si-OH) with hydroxyl and amino groups of DOX [61].

The MNPsFe_3_O_4_-SiO_2_-poly(NVCL-co-MAA)_33.45%_ nanocomposites showed a maximum release of approximately 55–60% at pH 7.4 for both temperatures (Figure 10a,b). At this pH value, the PMAA chains are swollen by the solvent, allowing the carboxylic groups from PMAA to form hydrogen bonds with the hydroxyl and amino groups from DOX and giving, as a result, a slow release rate of the drug. In other words, the strong electrostatic interaction between the positively charged DOX molecules and the negatively charged polymer chains did not allow the release of more DOX [59].

On the other hand, the drug-release profile at pH 5.8/25 °C for the MNPsFe_3_O_4_-SiO_2_-poly(NVCL-co-MAA) nanocomposite with 33 and 45% grafting showed a maximum release of DOX of 78 and 60% after 48 h, respectively. An increase in temperature to 37 °C leads to an increased release efficacy of 98 and 88% in the first 24 h for the nanocomposites with 33 and 45% grafting, respectively. The DOX release at pH 5.8/37 °C is higher than at pH 5.8/25 °C. This behavior is because, at acidic pH/25 °C, the copolymer chains are partially contracted (due to PMAA). In contrast, at 37 °C, the polymer chains from PNVCL and PMAA are contracted, allowing the release of more DOX. The electrostatic interactions between the DOX and polymeric chains from the nanocomposite are weaker at pH of 5.8 and 37 °C, obtaining a higher amount of released DOX [62,63]. Moreover, the release profiles in Figure 10 reveal that all the samples showed a sustained release pattern within the first 24 h.

Table 1 presents work on loading and releasing DOX of hybrid magnetic nanocomposites [59,62,64]. The results showed that the MNPsFe_3_O_4_-SiO_2_-poly(NVCL-co-MAA) nanocomposites exhibited minor DOX loading but higher DOX release, confirming the successful formulation process.

### 3.5. Hemolysis Assay

**Figure 11:**
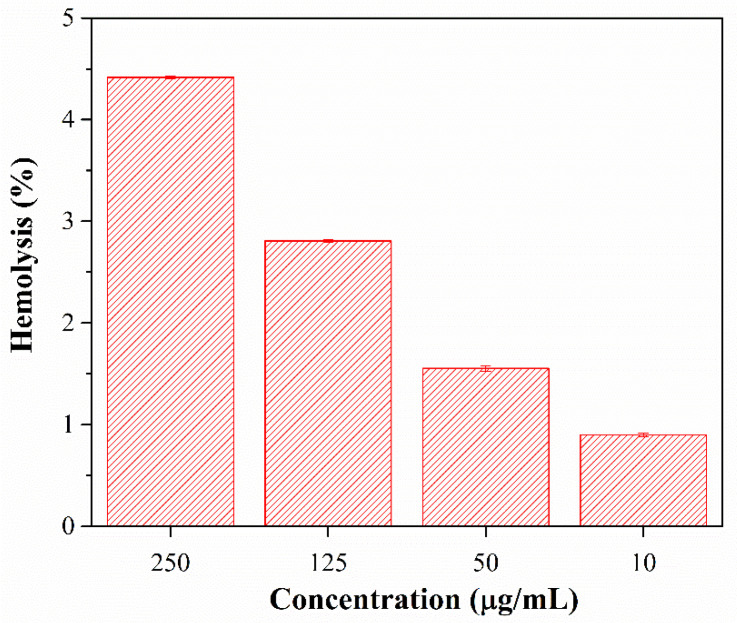
In vitro hemolysis (%) caused by different nanocomposite concentrations.

The hemolysis assay evaluates the hemoglobin release triggered by the breakdown of red blood cells due to chemical or mechanical stress. The material is considered highly hemocompatible if the hemolysis percentage is less than 5% [65]. As seen in Figure 11, the hemolysis percentage at concentrations of 10, 50, 125, and 250 μg/mL was less than 5%, indicating that the nanocomposite does not induce hemolysis. All the experiments showed no significant difference in the level of hemolysis (*p* < 0.05).

### 3.6. Release Kinetics

In order to investigate the mechanism of drug release, four kinetic models were fitted with DOX release data (Table 1). From Table 2, the best fit with the highest correlation coefficient is the Higuchi model (R^2^: 0.9594–0.9974), indicating that the release was controlled by diffusion. Using the Korsmeyer–Peppas equation, the data were fitted to determine the mechanism of drug release. The value of the drug release exponent (*η*) for the sample at pH 5.8/37 °C was 0.5357, indicating a non-Fickian drug-release mechanism in which the DOX is released by the relaxation of the polymer [66]. As Table 2 shows, the *η* is below 0.45 in the rest of the cases, indicating a Fickian diffusion mechanism of release.

## 4. Conclusions

In summary, we have successfully synthesized and characterized a pH- and temperature-sensitive nanocomposite based on MNPsFe_3_O_4_-SiO_2_ and grafted with poly(NVCL-co-MAA). According to previous studies, DOX release rates depend on pH and temperature. The nanocomposite showed faster release at pH 5.8 than at pH 7.4 and 37 °C than at 25 °C. The nanocomposite released 98% of DOX at pH 5.8 and 37 °C in the first 24 h. Furthermore, the nanocomposite showed non-hemolytic activity up to 250 mg/mL. The release data fit the Higuchi model in all cases, indicating diffusion controls the release of DOX. According to the results, it is inferred that the synthesized nanocomposite has a high potential to be used as a pH and temperature-responsive drug-delivery system.

## Figures and Tables

**Table 1:** Comparison of DOX loading and DOX release of hybrid magnetic nanocomposites reported by other research groups.

Research Group	Hybrid Magnetic Nanocomposites	DOX Loading (mg DOX/mg NC)	DOX Release at 37 °C	
			pH	(%)
This work	MNPsFe_3_O_4_-SiO_2_-poly(NVCL-co-MAA)_33%_	1.29 × 10^−2^	7.4	55
			5.8	98
	MNPs-Fe_3_O_4_-SiO_2_-poly(NVCL-co-MAA)_45%_	1.2 × 10^−2^	7.4	60
			5.8	88
Ma et al. [59]	MNPsFe_3_O_4_-SiO_2_-PAA	1.6 × 10^−2^	7.4	11
			5	69
Chang et al. [62].	MNPsFe_3_O_4_-MSN-poly(NIPAAm-co-MAA)	6.3 × 10^−2^	7.4	7.2
			6.5	37
			5	80
Pon et al. [64]	MNPsFe_3_O_4_-SiO_2_-(CS-PNIPAAm)	7.4 × 10^−2^	7.4	26
			4	57

**Table 2:** In vitro release kinetic values of DOX from MNPsFe_3_O_4_-SiO_2_-poly(NVCL-co-MAA)_33%_ nanocomposite at different pH and temperature values.

Kinetic Model	Zero-Order		First-Order		Higuchi		Korsmeyer-Peppas		
Parameters	*K*_0_	R^2^	*K*_1_	R^2^	*K_H_*	R^2^	*K_K_*	*η*	R^2^
pH 7.4, 25 °C	0.6171	0.9730	0.0097	0.9122	6.2459	0.9974	0.8998	0.4403	0.9971
pH 7.4, 37 °C	0.7390	0.9707	0.0095	0.8963	5.9874	0.9854	1.2129	0.2901	0.9775
pH 5.8, 25 °C	1.7640	0.9218	0.0174	0.8016	11.903	0.9768	1.2594	0.4194	0.9744
pH 5.8, 37 °C	8.3970	0.8619	0.0608	0.7514	33.854	0.9594	1.5212	0.5357	0.9587

## Data Availability

Not applicable.

## Supplementary Materials

The following supporting information can be downloaded at: https://www.mdpi.com/article/10.3390/polym15040968/s1, Figure S1. FTIR spectra of MNPsFe_3_O_4_-SiO_2_-poly(NVCL-co-MAA) nanocomposite with different monomer ratios (NVCL:MAA). Figure S2. Cumulative DOX release profile for MNPsFe_3_O_4_-SiO_2_.
