# Supplementary material for: Synthesis and Characterization of a pH- and Temperature-Sensitive Fe3O4-SiO2-Poly(NVCL-co-MAA) Nanocomposite for Controlled Delivery of Doxorubicin Anticancer Drug
Source: Polymers (Basel). 2023 Feb 15;15(4):968. doi: 10.3390/polym15040968 (PMC9963235; doi:10.3390/polym15040968)
Supplement: Supplementary file 1 [file polymers-15-00968-s001.zip › polymers-2186618-supplementary.pdf]

Supplmentary Materials

Synthesis and Characterization of a pH- and Temperature-Sensitive Fe_3O_4-SiO_2-Poly(NVCL-co-MAA) Nanocomposite for Controlled Delivery of Doxorubicin Anticancer Drug

Jorge Luis Sánchez-Orozco [1], Héctor Iván Meléndez-Ortiz [2], Bertha Alicia Puente-Urbina [1], Oliverio Santiago Rodríguez-Fernández [1], Antonia Martínez-Luévanos [3] and Luis Alfonso García-Cerda [1,*]

1. Centro de Investigación en Química Aplicada, Departamento de Materiales Avanzados, Blvd. Enrique Reyna Hermosillo #140, Saltillo 25294, Mexico
2. CONACyT-Centro de Investigación en Química Aplicada, Blvd. Enrique Reyna Hermosillo #140, Saltillo 25294, Mexico
3. Facultad de Ciencias Químicas, Universidad Autónoma de Coahuila, Blvd. V. Carranza s/n, Saltillo 25280, Mexico

* Correspondence: luis.garcia@ciqa.edu.mx; Tel.: +52-8444389830

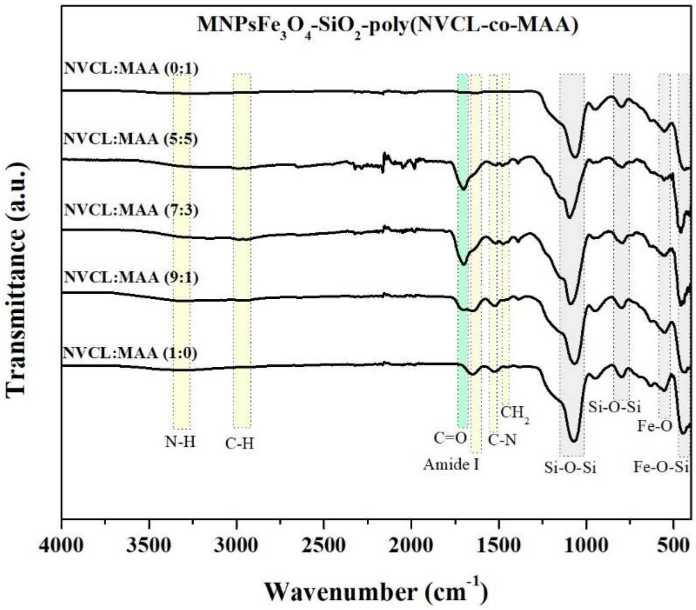

Figure S1. FTIR spectra of MNPsFe_3O_4-SiO_2-poly(NVCL-co-MAA) nanocomposite with different monomer ratios (NVCL:MAA).

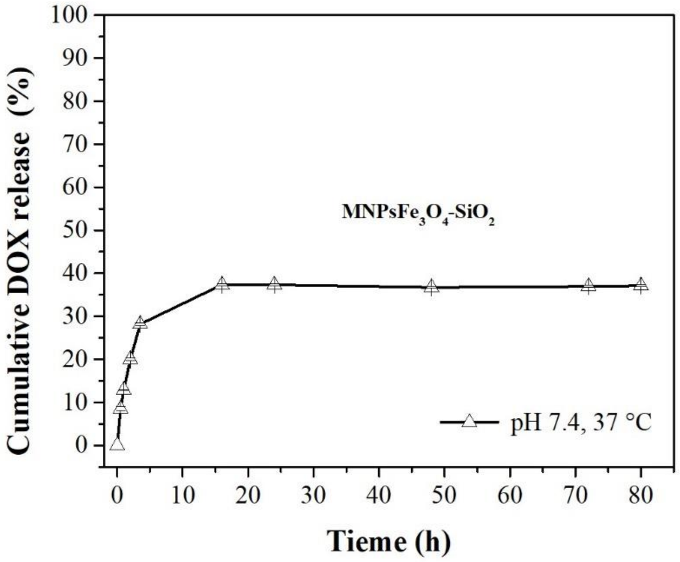

Figure S2. Cumulative DOX release profile for $MNPsFe_3O_4$-SiO_2.
